# What Can We Learn for Future Integrated Care Models in Long Term Care Facilities After the COVID-19 Emergency? Lessons From an Observational Study in Catalonia

**DOI:** 10.5334/ijic.8597

**Published:** 2025-03-19

**Authors:** Mireia Massot Mesquida, Miquel À. Mas, Rosa García-Sierra, Sara Pablo Reyes, Ramón Miralles Basseda, Xavier Vallès, Irene Garcia, Sara Rodoreda, Mar Isnard Blanchart, Maria Josep Ulldemolins, Ricard Peiró Navarro, Susana Morales, Boris Trenado, Yolanda Ordorica, Marta Expósito Izquierdo, Maria José Pérez Lucena, Nemesio Moreno, Montserrat Teixidó Colet, Norma Henríquez, Joaquim Verdaguer Puigvendrelló, Josep Maria Bonet, Núria Prat, Eduard Lozano, Rosa López, Oriol Estrada, Jordi Ara

**Affiliations:** 1 Direcció d’Atenció Primària Metropolitana Nord, Institut Català de la Salut, Catalonia, Spain; 2 Grup de Recerca Multidisciplinari en Salut i Societat (GREMSAS), Unitat de Suport a la Recerca Metropolitana Nord, Institut Universitari d’Investigació en Atenció Primària Jordi Gol (IDIAP Jordi Gol), Mataró, Spain; 3 Department of Geriatrics, Hospital Universitari Germans Trias i Pujol, Badalona, Spain; 4 Direcció Clínica Territorial de Cronicitat Metropolitana Nord, Institut Català de la Salut, Barcelona, Catalonia, Spain; 5 International Health Program, Regió Sanitària Metropolitana Nord, Institut Català de la Salut, Badalona, Spain; 6 Institut per la Recerca en Ciències en Ciències de la Salut Germans Trias i Pujol, Badalona, Spain; 7 Direcció d’Organització i Sistemes d’Informació Metropolitana Nord. Institut Català de la Salut, Barcelona, Catalonia, Spain; 8 Gerència Territorial Metropolitana Nord, Institut Català de la Salut, Barcelona, Catalonia, Spain

**Keywords:** Long Term Care facilities, COVID-19, integrated care models

## Abstract

**Introduction:**

The healthcare response to the COVID-19 pandemic in long term care facilities (LTCF), constitutes one of the challenges faced by governments and institutions worldwide. Our aim was to analyze the facilitators and barriers of this response, for the future integrated care model in these facilities.

**Methods:**

From a retrospective observational study, we present the experience and lessons learned of the implementation of an integrated response at the meso level in LTCF for older people and for people with physical and mental conditions in the North Metropolitan area of Barcelona, in Catalonia, during the COVID-19 pandemic.

**Results:**

We analyzed the care provided to 13,369 institutionalized people. The major facilitating points were: the adaptation of proactive care teams, the creation of a tool to improve communication with institutions, and the management of epidemiological data for planning collaboration between different actors. Main barriers were not including users and family members views in the response adaptation, and the lack of LTCF resources to respond to changing needs.

**Conclusions:**

Increasing proactivity and adapting interventions based on updated information were key to minimize infections and mortality. Improving the communication and the collaboration between actors, and people involvement in the response planning, need to be considered for the future.

## Introduction

In recent decades, the epidemiology of chronic disease and the increased dependency of the European population has resulted in greater use of the resource of institutionalization in long-term care facilities (LTCF) [1,2]. As a result, the traditional profile of institutionalized patients has changed, and there is now greater complexity due to various factors such as older age, high prevalence of frailty, the accumulation of chronic diseases, high consumption of health resources, and an increase in palliative care needs [3,4,5]. A study conducted from 2011 to 2017 on a population of 93,038 residents of nursing homes in Catalonia (in South-Western Europe) reported an increase in mean age from 83 to 87 years and an increase in annual mortality from 11.7% to 20.5% [6]. On other issue, LTCF have been attended other populations with high vulnerability, such as young people with diverse mental and physical chronic conditions who face similar challenges during institutionalization than frail older populations [7]. Traditionally, the response to social and health needs in these facilities has been very heterogeneous across different regions, since it has been based on a fragmented, reactive model to the needs that arise from the decompensation of chronic diseases or the progression of disabling pathologies, rather than in covering individuals needs.

The outbreak of the COVID-19 pandemic in early 2020 posed an enormous challenge for health and social systems. Not only did LTCF present the ideal conditions for the airborne transmission of a virus, but older people with high frailty rates were at greater risk of developing acute COVID-19, and the complexity of controlling the disease in these facilities was very high. In the early stages, aggressive transmission was observed, especially through asymptomatic patients, and there was high incidence and high mortality (that was higher in institutions than in the community) [8]. The initial response to the crisis in LTCF was limited by various epidemiological and organizational factors such as infection-control strategies (residents’ isolation or transmission protection, between others), and population factors related to mortality [9,10,11,12]. This unprecedented situation in the history of recent medicine overwhelmed professionals, institutionalized residents and their families, and had a major impact, especially on the care of people who were already nearing the end-of-life [13,14].

### Overview of the Catalan health and social care system

The Catalan healthcare model guarantees access to healthcare for the entire population residing in Catalonia (North-East of Spain). It is mostly financed through taxes and is mostly public, although private centers can be offered through agreements with the public administration. The healthcare administration is decentralized, with the different territorial health regions managing resources according to the characteristics of their population, allowing management to be tailored to their needs. It has been developed an integrated care model that seeks greater coordination between health and social services to provide more comprehensive care to people with complex needs [15]. In this way, there is a holistic approach to health, from public health to primary and community care, hospital care, intermediate care, mental health care, and also care from the social services. Universal healthcare is provided by the primary care units of the public health system, which are responsible for the home care of vulnerable people, regardless of their place of residence. On the other hand, the majority of LTCF are privately owned.

### Rationale and aim of this study

In the North Metropolitan area of Barcelona, the Directorate of Primary Care, provides care coverage to an assigned population of up to 1,9M inhabitants, of which, during the Covid-19 pandemic, more than 10,000 were institutionalized people. A total of 64 Primary care teams (PCT) played a crucial role in adapting the care model to the pandemic and relied on the learning-by-doing strategy [16] in its response to the COVID-19 in LTCF. The aim of our study was to describe the evolution of organizational changes during the COVID-19 emergency and their interaction with outcomes (infection spread and mortality) across different waves, to understand the complexity factors in caring for these populations, and to define key aspects to adapt future integrated care models in LTCF based on the lessons learned.

## Methods

### Study design, period, population and variables

This is a retrospective observational study. The observation period was between April 2020 and November 2021. The study setting was the Metropolitana Nord Directorate of Primary Care of the Institut Català de la Salut (Catalan Health Institute), main public provider of Primary Care in our country. We included data from all of the institutionalized people attended during the study period were included. The main study variables were the incidence of COVID-19 and mortality rates in residents, and incidence in LTCF workers in each wave and for the type of facility. To objectify the evolution of the infection and mortality rates of both residents and professionals in relation to the various interventions performed, anonymized data was extracted from computerized clinical records of the institutionalized population and workers assigned to our primary care centers.

### Description of the methodology applied for the pandemic response

A single action strategy was developed for the 64 PCT that provide care coverage to the institutionalized population in our area. Our learning-by-doing methodology comprised three basic elements:

- Theoretical knowledge: A multidisciplinary task force formed by experts in the care of institutionalized patients was established. It included primary care physicians and nurses, geriatricians, preventive medicine physicians, and pharmacists. Its mission was twofold: epidemiology and care. The team proposed and planned actions at consensus meetings. To guarantee uniform implementation of the model throughout the region, the task force considered regional diversity and scope. It established and unified strategic lines of prevention, diagnosis and clinical management of institutionalized patients affected by COVID-19 for the entire health region.
- Practical implementation of the knowledge acquired: The pre-existing PCT structure was bolstered. These teams provided direct care at the nursing facilities around the clock (24/7) to guarantee continuity of care. The deployment of these teams was homogeneous throughout the region and thus facilitated the ongoing detection of needs, strengths and weaknesses of the measures taken in the nursing facilities. Each team attended the facilities allocated from its territory (mainly between 1-5 facilities of different size depending on the location) in a daily basis, to address the challenges faced by residents in every institution.
- Modification of the model based on lessons learned: A series of technical measures were defined to monitor the impact of the actions carried out to learn from mistakes and modify the actions over time. In this regard, one key task was the monitoring of clinical and epidemiological data through the regional management’s specific information system tools.

### Analysis

We analyzed the data in two parts: the quantitative epidemiologic data and the narrative of the implementation of the learning-by-doing process. The COVID-19 incidence was calculated using the number of new cases divided by the population at risk, and the mortality rate was calculated using the number of deaths divided by the total population. Moreover, we defined a trajectory of evolution of the pandemics in LTCF by combining actions from the implementation process and epidemiological results.

### Ethics

The study protocol was approved by the Ethics and Clinical Research Committee of the Jordi Gol Institute for Primary Care Research (IDIAP) (Code: 21/142-PCV).

## Results

The total population included in the analysis comprised 13,369 institutionalized patients and 7,661 professionals, from 169 care homes and in 26 LTCF for people with physical and/or mental conditions.

### A. Summary of epidemiological data

Table 1 shows the incidence of COVID-19 and the distribution of patients by facility type. The high incidence in convents and care homes for the elderly during the first waves stands out in comparison with later waves.

**Table 1:** Distribution of the incidence of COVID-19 in institutionalized patients by nursing facility type.


INCIDENCE OF COVID PER 1,000 RESIDENTS														

			WAVES AND VALEY PHASES											

		NUMBER OF RESIDENTS	1ST WAVE	VALLEY PHASE 1	2ND WAVE	VALLEY PHASE 2	3D WAVE	VALLEY PHASE 3	4TH WAVE	VALLEY PHASE 4	5TH WAVE	VALLEY PHASE 5	6TH WAVE	TOTAL

NURSING FACILITY TYPE	Nursing facilities for the elderly	**11,550**	189.44	82.68	90.74	11.17	73.07	1.30	1.56	1.56	27.97	4.85	114.29	**598.61**

	Mental health center	**203**	59.11	14.78	19.70	0.00	14.78	4.93	0.00	4.93	49.26	9.85	68.97	**246.31**

	Center for disabled people	**1228**	140.88	92.83	134.36	31.76	123.78	8.14	0.00	0.00	53.75	0.81	96.09	**682.41**

	Youth home	**297**	0.00	26,94	43.77	3.37	23.57	6.73	3.37	3.37	77.44	10.10	144.78	**343.43**

	Center for people at risk of social exclusion	**55**	109.09	18.18	36.36	0.00	54.55	18.18	0.00	0.00	0.00	0.00	163.64	**400.00**

	Convent	**36**	194.44	194.44	0.00	0.00	0.00	0.00	0.00	0.00	0.00	0.00	55.56	**444.44**

**TOTAL**		**13369**	**178.47**	**81.38**	**92.15**	**12.64**	**75.47**	**2.17**	**1.42**	**1.50**	**31.57**	**4.64**	**112.65**	**594.06**


Table 2 shows the mortality rate of patients with a confirmed COVID-19 diagnosis, both in total and based on the amount of time since diagnosis. A decrease in mortality compared to the first wave can be observed. The mortality rates of the first wave are significant both just after diagnosis and later on.

**Table 2:** Mortality rate of institutionalized patients with confirmed COVID-19 diagnosis.


COVID MORTALITY RATE PER 1,000 RESIDENTS												

	1ST WAVE	VALLEY PHASE 1	2ND WAVE	VALLEY PHASE 2	3D WAVE	VALLEY PHASE 3	4TH WAVE	VALLEY PHASE 4	5TH WAVE	VALLEY PHASE 5	6TH WAVE	TOTAL

RESIDENTS WITH COVID DIAGNOSIS	97.84	32.31	30.37	3.89	19.37	0.22	0.37	0.30	6.88	0.22	2.69	**194.48**

≤30 DAYS SINCE DIAGNOSIS	57.90	11.89	11.89	1.12	6.96	0.00	0.07	0.15	3.74	0.00	2.02	**95.74**

>30 DAYS SINCE DIAGNOSIS	39.94	20.42	18.48	2.77	12.42	0.22	0.30	0.15	3.14	0.22	0.67	**98.74**


Table 3 shows the incidence and distribution of COVID-19 in workers by LTCF type. The high incidence rate in nursing facilities workers in the last wave analyzed is noteworthy.

**Table 3:** Distribution of the incidence of COVID-19 in workers by nursing facility type.


INCIDENCE OF COVID PER 1,000 WORKERS														

			WAVES AND VALEY PHASES											

		NUMBER OF WORKERS	1ST WAVE	VALLEY PHASE 1	2ND WAVE	VALLEY PHASE 2	3D WAVE	VALLEY PHASE 3	4TH WAVE	VALLEY PHASE 4	5TH WAVE	VALLEY PHASE 5	6TH WAVE	TOTAL

NURSING FACILITY TYPE	Nursing facilities for the elderly	**5970**	58.96	47.91	76.55	7.87	64.49	5.19	7.20	1.51	64.66	17.76	204.86	**556.95**

	Mental health center	**144**	48.61	20.83	20.83	0.00	13.89	0.00	0.00	0.00	41.67	13.89	194.44	**354.17**

	Center for disabled people	**1407**	24.16	24.16	78.89	9.24	61.83	2.84	4.26	2.13	65.39	14.93	188.34	**476.19**

	Youth home	**99**	0.00	0.00	101.01	0.00	10.10	0.00	0.00	0.00	70.71	10.10	242.42	**434.34**

	Center for people at risk of social exclusion	**28**	0.00	35.71	71.43	0.00	71.43	0.00	35.71	35.71	107.14	0.00	214.29	**571.43**

	Convent	**13**	0.00	0.00	384.62	0.00	0.00	76.92	0.00	0.00	0.00	0.00	230.77	**692.31**

	**TOTAL**	**7661**	**51.30**	**42.29**	**76.75**	**7.83**	**62.26**	**4.70**	**6.53**	**1.70**	**64.48**	**16.97**	**202.19**	**537.01**


### B. Summary of actions from the implementation process

**Figure 1:**
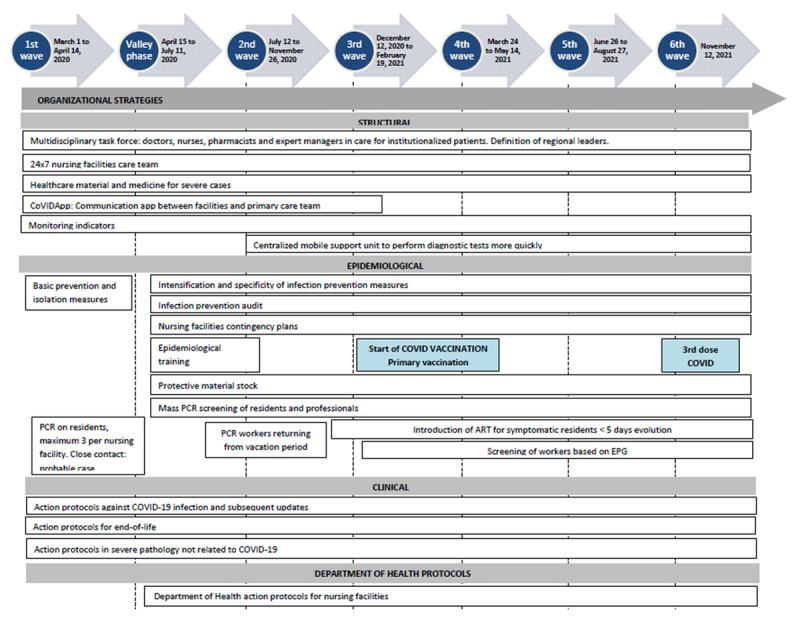
Main characteristics of the actions from the operational pandemic response. PCR: Polymerase Chain Reaction. EPG: Epidemiological Potential Growing. Trend indicator of the risk of relapse. ART: Rapid antigen test.

Different phases in the evolution of the model can be discerned based on the actions taken at various stages of the main waves of the pandemic (Figure 1).

#### B1. First wave: identification of organizational needs (spring 2020)

We identified organizational needs in order to establish action protocols common to all LTCF in the region. The multidisciplinary task force focused its efforts on creating, updating and guaranteeing the rapid dissemination of consensus-based protocols for managing residents with COVID-19 and reinforcing the protocol for managing emergency end-of-life situations, in anticipation of the high mortality previously described in other countries. General measures for preventing infection were established. During the first wave, it became clear that respiratory symptoms in residents as the indicator for the activation of prevention measures for infection and clinical complications was too narrow. At the same time, a 24/7 care strategy with a reinforced structure was designed for all LTCF in the region to ensure there would be a group of professionals responsible for continuous care. Moreover, the need for both the provision of medication for severe cases and/or end-of-life situations, and protective equipment for health professionals was detected and met.

Regarding communication needs, a mobile app (CoVIDApp) [17] was adapted to improve communication between professionals at LTCF and primary care professionals. The LTCF managers used this app to report new daily cases and the evolution of cases already detected—which activated the primary care team for urgent care when necessary—, PCR results, and clinical variables to monitor residents remotely. It was also possible to record the status of workers and detect facilities at risk of not being able to respond to the situation adequately. A control panel was created with the tool Business Objects to specifically monitor the response at each center and region in our sphere of influence.

Strategic alliances with all the care levels and providers involved was a key element in applying measures established through institutional policy, as well as local care requirements: a) for the epidemiological strategy, alliances between health and social institutions were focused on establishing effective measures to prevent and control the spread of infection in the facilities. These measures were determined by the institutional policy framework (policy level) to be applied by the health provider (provider level). All the mandatory measures established by the institutional policy gave us common protocols for all areas: policy, primary care and LTCF, which facilitated coordination between them; b) for the care strategy, an attempt was made to involve the various actors (from health and social sectors) in the response through a comprehensive care model that could be adapted to the different realities of institutionalized patients—whether affected by COVID-19 or not—, existing needs, and the evidence available.

#### B2. Valley phase: adaptation of the model based on lessons learned (summer 2020)

Thanks to the support of the PCT providing direct care at the LTCF, the underlying factors that determined whether facilities were at risk of being unable to respond to the pandemic were detected and analyzed. Along with results from the CoVIDApp, these factors helped us modify the action strategy based on the following difficulties:

- Snowball effect: The facilities that did not adequately follow protection and prevention measures had a higher infection rate, both in residents and professionals. This resulted in a shortage of professionals who could adequately care for residents, and thus, worse prognosis for patients once infected. As a corrective measure, the prevention strategy was reinforced: an audit was designed with 12 mandatory items that nursing facilities had to comply with for the correct prevention of infection.
- There was a turning point when it was demonstrated that there were positive residents who were asymptomatic and that, on some occasions, the symptoms were not always respiratory in nature. Corrective proactive responses were established, consisting of the mass screening of all residents and professionals at the center when one resident tested positive. The recommendation was made to screen residents when they returned from a stay outside the facility, as well as workers upon return from a vacation period.
- To quickly respond to the demand for mass screening, a mobile regional unit was structured to help PCT performing them.

On the other hand, the Department of Health, Public Health and the Social Welfare established a series of mandatory common protocols to be applied in all areas, both at the level of healthcare providers and at the level of LTCF, based on emergent evidence. on the management of COVID-19 infection.

#### B3. Second and third waves (winter 2020–spring 2021)

Based on accumulated knowledge from previous phases, we applied proactive measures to prevent infection during the second wave. The aim was to prevent the virus from entering facilities and, if it did manage to get in, prevent or minimize transmission inside.

- The availability of the rapid antigen test changed the paradigm of infection in LTCF. Rapid tests made it possible to quickly diagnose (15 minutes) patients with symptoms compatible with COVID-19, thus enabling an immediate sweep of both workers and residents at the nursing facilities with PCR tests in search of asymptomatic positive patients so that sectorization and intervention in the residence could be performed quickly and early on.
- Another proactive measure established by the regional government was focused on preventing the virus from entering facilities. Both residents and workers underwent PCR screening when they returned to the nursing facilities after a vacation period of more than 15 days.

During the third wave, as evidence on the management of COVID-19 emerged and new evidence on various pharmacological treatments appeared, the action protocols were updated. Therefore, two new prevention strategies were established:

- Given the high prevalence of infection in the community, periodic screening measures were also activated for LTCF workers (both permanent and temporary workers) since the data indicated that the virus was initially entering the residences through them. If this occurred at an at-risk LTCF, the care team supervised the implementation of the process.
- Rapid communication channels, between PCT and LTCF, were established for screening results if a worker or resident tested positive.
- The irruption of vaccination: with the initiation of the mass vaccination strategy for institutionalized residents, in December 2020, a huge leap was made in epidemiological control and the minimization of the risk of complications in terms of protecting this population.

#### B4. Fourth wave and later (from spring 2021)

The actions consolidated during the previous waves were maintained and the various clinical management protocols were updated based on the evidence and introduction of new treatments. In September 2021, the vaccination of institutionalized patients with the third booster shot began, which alongside the other preventive and organizational measures, constituted a more proactive and coordinated care response.

#### C. Interaction between implemented actions and epidemiological results

**Figure 2:**
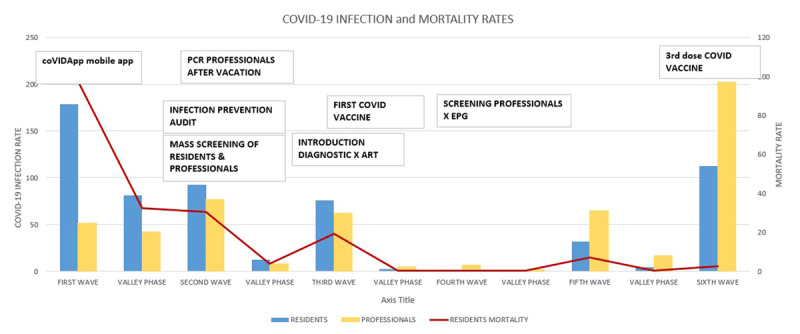
Impact of different measures adopted in our territory to favor control of COVID-19 infection and mortality rates. ART: Rapid antigen test. EPG: Epidemiological Potential Growing. Trend indicator of the risk of relapse.

Figure 2 presents the evolution of infection and mortality rates alongside the implementation of the measures adopted in the various phases.

The positive evolution of the COVID-19 infection rate in our population over the course of the various waves could be attributed to multiple conditioning factors, although the data suggest a relation between increased prevention measures and decreased infection rate in residents over time. In this sense, it was key, during the first wave, to implement a digital tool (as CoVIDApp) to have direct information, of both residents and workers, from LTCF. Main preventive actions, that reduced COVID-19 infections after the first wave, were mass screening after detecting a positive case, which allowed us to detect asymptomatic cases and stop the spread of the virus once it had entered the facilities, and infection prevention audits, that reflected the response capacity of the LTCF if an infection were to break out. Mass screening of the professionals, directly caring for residents, became mandatory in periods after the second wave, and from then on, the detection of asymptomatic positive workers was higher than in the first two waves. The irruption of the vaccination in the third wave, was a central action to reduce mortality, complementing the other preventive actions implemented during the previous waves.

Lastly, the drastic reduction in the mortality rate of residents in later waves as compared to the first and second waves, falls in line with the reduced infection rate of both residents and workers, which corroborates that the best strategy is to prevent infection. A decrease in the specific mortality of infected residents is also observed, which can be explained by other cofactors that were directly or indirectly controlled, such as high viral load due to risk contacts, delayed diagnosis, and reinfection.

## Discussion

The COVID-19 pandemic has had a negative impact on institutionalized people in our population and worldwide, and all lessons learned from the emergency are needed to improve the integration of care, from health and social sectors, for the future. The impact on LTCF populations has been bigger than in other populations due to the great epidemiological and clinical vulnerability of this population as our group has demonstrated in various studies [10,11,12], leading to an excess of mortality [18]. This situation obliged institutions to study and apply different strategies to reduce the impact of both the infection and mortality rate in these patients. The actions adopted in our strategy were comparable with measures used in other territories. In a systematic review published in 2022, from a Spanish group, they were found key strategic, operational and support processes [19], linked to good practices, similar to ours, incorporated into LTCF processes during the pandemic. In a rapid mapping review of international evidence performed by Byrd et al. [20], several domains and actions were identified such as: LTCF multifaceted interventions, focused on preventing or controlling Covid-19 infections, data and information and communication technology use, policy and governance actions in the social sector, and identifying possible targets for policies and interventions (including ownership structures, quality of services, and staffing policies). The fact that proactive actions, such as mass screening, were key to prevented the entry and spread of the virus in the facilities, as was later corroborated [21,22].

Based on the analysis of our work, we identified facilitators and barriers of the integration of the health and social care response, that can be useful for care planners, clinicians and users, in order to improve the integration of care provision in LTCF in the future.

### I. Facilitators of the integrated care response

#### Impact of a preventive response led by Primary Care Teams

Through the collaboration between the actions from our PCT and the other LTCF actors, we noticed a relation between increased preventive measures and decreased infection rate and mortality in residents over time, as identified in the lessons learned article by Fang et al. [23]. In this sense, a Dutch study, by van Tol et al., reinforced the impact of outbreak teams, including clinicians, managers, and policy advisers to coordinate COVID-19 infection prevention and control, in LTCF [24].

#### The importance of communication between health and social actors

The adaptation of information technologies to improve communication between professionals, by using a mobile App, was found a crucial tool at a time of changing information and great uncertainty. Various groups used simple tools to share information, and they have been considered key elements in the integration of services for institutionalized people [19].

#### Continuous adaptation to changing needs

One of the decisive elements during the COVID-19 pandemic operations was first-hand knowledge and analysis of the information available on the changing reality of facilities. Having a specific information system that was updated on a daily basis was extremely useful for planning the emergency response. In this sense, it is essential to understand the changing needs of this population to plan integrated care policies in LTCF at the micro, meso, and macro level.

#### Evolution of the care model from reactivity to proactivity

Especially in the early stages when there was much uncertainty and little knowledge of the disease, the pandemic demanded very intense and completely proactive intervention from Primary Care professionals based on the information received from LTCF teams. This increased intensity of healthcare opened the doors to greater joint work between different professionals. In this sense, we believe it is important to reinforce and promote the collaboration between professionals from the health and social sectors, in the context of care provision in LTCF.

#### The key role of strategic alliances and good governance

Another key point in the initial response to the pandemic was the alignment of objectives in the collaboration of various actors of the health and social systems. The different departments (health and social) of our regional government, and the social care and healthcare providers had to establish a common working path to respond to the emergency. In this sense, there were many micro barriers to the effective integration of the responses, which should make us reflect on what are the best ways to pose policies and manage integrated care in the future.

### II. Barriers to an integrated care response

It is very important to consider the limitations of focusing the model on the health needs of vulnerable populations, since, although they represent a significant part of the needs to be addressed, there are other key elements to consider, including socio-familiar needs or factors related to LTCF structure, organization and resources. An Italian study, by Notarnicola et al. found fragmentation, challenges regarding coordination with the healthcare sector, questioning the vocation of the services, and inadequate allocation of resources, between others, as main weaknesses of the LTC system to response to all needs of these vulnerable populations during the COVID-19 emergency [25].

We list different elements below, as they should be taken into consideration for future models.

#### Extreme vulnerability of the institutionalized population

Current care services for people with extensive health and social needs due to frailty, disability and complex disease needs (including older and young populations) should consider the importance of developing personalized care plans adapted each individual’s needs (different background, and physical and mental conditions) and that can be modified continuously in changing situations. After the COVID-19 pandemic, the Government of Catalonia, as other governments worldwide, have developed specific plans to improve the care of vulnerable populations living in LTCF [26].

#### Lack of including the opinion of the people being served

In our experience, there was no focus on the needs of individuals when planning the epidemiological responses. In this sense, loneliness and lack of stimulation have been identified as one of the weaknesses of care in LTCF during the pandemic [27].

#### Lack of considering relatives needs

The residents’ loved ones have also suffered during the pandemic, especially due to restrictions on visits and companionship, and the epidemiological demands, which were prioritized over personal needs. Given the large number of people who are institutionalized due to disabling conditions, it is especially important to understand their concerns and needs.

#### Lack of LTCF resources

The pandemic has revealed the need to guarantee many continuous resources to meet the needs that arise from the health crisis. This has highlighted the fact that many facilities do not have the resources required to cover the needs of the people served. This issue must be corrected to guarantee good social and health care [25].

#### Limitations and biases of the study

Our work urges from an observational study analysing of the impact of a local strategy in a single territory in the metropolitan region of Barcelona, in South-western Europe. Main conclusions of our work need to be considered locally, and could not be representative of the reality of other territories. Moreover, the discussion of facilitators and barriers of the integrated care response was constructed based on the identification of main issues identified by our group, and it could not be representative of the views of the other actors linked to the response. In this sense, conclusions and discussions should be interpreted and adapted based on the context of other views and experiences. Unfortunately, LTCF people with lived experience have not been involved in the underlying study or in preparation of the manuscript.

## Lessons learned

Our experience, an emergency approach at the meso level in the South of Europe, of adapting the health response to a vulnerable institutionalized population at risk during the pandemic has yielded several lessons.

- The model in use up until the pandemic had limited capacity for planning and proactivity and focused health interventions on reactivity.
- For the emergency response, the availability of technological tools for communication between actors and for data analysis was a key element in adapting to the changing needs of the population served.
- The new model must have a deep focus on the provision of integrated care, not only health but social, with the availability of enough resources to respond to the vulnerability of the different institutionalized populations.
- Alliances between different actors must have the common objective of providing a response centered on people needs.

## Conclusion

The COVID-19 pandemic has posed a challenge for the health and social system in our region and throughout the world, with LTCF being one of the settings that has most required adapted strategies. Our response in the metropolitan area of Barcelona has revolved around the change from a reactive to a proactive response and has relied on key elements for a more integrated model, such as the use of information technologies and the consensus of responses between different actors. The lessons learned and the main limitations detected, lack of person-centred approaches in the care planning, should be taken into account for improving the current care models of LTC and to advance to the integrated care models implementation.

## References

[1] European Commission, Eurostat. Healthcare resource statistics – beds, August 2020. [webpage on internet]. [cited 2023 August 31]. Available from: https://ec.europa.eu/eurostat/statistics-explained/index.php?title=Healthcare_resource_statistics_-_beds#Long-term_care_beds_in_nursing_and_residential_care_facilities

[2] World Health Organization. Number of nursing and elderly home be–s – European Health Information Gateway [webpage on internet]. [cited 2023 August 31]. Available from: https://gateway.euro.who.int/en/indicators/hfa_491-5101-number-of-nursing-and-elderly-home-beds/visualizations/#id=19556

[3] Gordon AL, Franklin M, Bradshaw L, Logan P, Elliott R, Gladman JRF. Health status of UK care home residents: a cohort study. Age Ageing. 2014; 43: 97–103. DOI: 10.1093/ageing/aft077 23864424 PMC3861334

[4] Li Q, Zheng NT, Temkin-Greener H. End of Life Quality of Care Among Long-Term Nursing Home Decedent Residents With and Without Dementia. J Am Geriatr Soc. 2013; 61: 1066–1073. DOI: 10.1111/jgs.12330 23772891 PMC4122312

[5] García-Gollarte JF, Montero García-Andrade M, Santaeugenia-González SJ, Solá Hermida JC, Baixauli-Alacreu S, Tarazona Santabalbina FJ. Risk Factors for Mortality in Nursing Home Residents: An Observational Study. Geriatrics (Basel). 2020; 5(4): E71. DOI: 10.3390/geriatrics5040071 PMC7709674 33050016

[6] Amblàs-Novellas J, Santaeugènia SJ, Vela E, Clèries M, Contel JC. What lies beneath: a retrospective, population-based cohort study investigating clinical and resource-use characteristics of institutionalized older people in Catalonia. BMC Geriatr. 2020; 20(1): 187. DOI: 10.1186/s12877-020-01587-8 32487082 PMC7265641

[7] Howard EP, Martin L, Heckman GA, Morris JN. Does the Person-Centered Care Model Support the Needs of Long-Term Care Residents With Serious Mental Illness and Intellectual and Developmental Disabilities? Front Psychiatry. 2021: 12: 704764. DOI: 10.3389/fpsyt.2021.704764 34867509 PMC8632811

[8] Cases L, Vela E, Santaeugènia Gonzàlez SJ, Contel JC, Carot-Sans G, et al. Excess mortality among older adults institutionalized in long-term care facilities during the COVID-19 pandemic: a population-based analysis in Catalonia. Front Public Health. 2023; 11: 1208184. DOI: 10.3389/fpubh.2023.1208184 37732085 PMC10507684

[9] Arons MM, Hatfield KM, Reddy SC, Kimball A, James A, Jacobs JR, et al. Presymptomatic SARS-CoV-2 Infections and Transmission in a Skilled Nursing Facility. N Engl J Med. 2020; 382(22): 2081–2090. DOI: 10.1056/NEJMoa2008457 32329971 PMC7200056

[10] Suner C, Ouchi D, Mas MÀ, López Alarcón R, Massot Mesquida M, Negredo E, et al. A retrospective cohort study of risk factors for mortality among nursing homes exposed to COVID-19 in Spain. Nat Aging. 2021; 1: 579–584. DOI: 10.1038/s43587-021-00079-7 37117802

[11] Mas MÀ, Mesquida MM, Miralles R, Soldevila L, Prat N, Bonet-Simó JM, et al. Clinical Factors Related to COVID-19 Outcomes in Institutionalized Older Adults: Cross-sectional Analysis from a Cohort in Catalonia. J Am Med Dir Assoc. 2021; 22(9): 1857–1859. DOI: 10.1016/j.jamda.2021.07.004 34375654 PMC8289628

[12] Soldevila L, Prat N, Mas MÀ, Massot M, Miralles R, Bonet-Simó JM, et al. The interplay between infection risk factors of SARS-CoV-2 and mortality: a cross-sectional study from a cohort of long-term care nursing home residents. BMC Geriatr. 2022; 22(1): 123. DOI: 10.1186/s12877-022-02779-0 35164680 PMC8842505

[13] Amblàs-Novellas J, Gómez-Batiste X; professionals and organizations that have participated in the consensus. Clinical and ethical recommendations for decision-making in nursing homes in the context of the COVID-19 crisis. Med Clin (Engl Ed). 2020; 155(8): 356–359. DOI: 10.1016/j.medcle.2020.06.015 33024824 PMC7528908

[14] Amblàs-Novellas J, Martínez-Gómez R, Blasco-Rovira M. [Palliative care in nursing homes during the COVID-19 pandemic (or when coronavirus knocked at the door of the system’s most vulnerable setting)]. Med Paliat. 2020; 27(3): 234–241. [in Spanish]. DOI: 10.20986/medpal.2020.1169/2020

[15] Generalitat de Catalunya. Ministry of Health. Catalan model of care for people with frailty, complex chronic (CCP) and advanced chronic (ACP) conditions. Barcelona 2021. [cited 2023 31 August]. Available from: https://scientiasalut.gencat.cat/bitstream/handle/11351/7007/bases_conceptuals_model_atencio_persones_fragils_cronicitat_complexa_avancada_2020_ang.pdf?sequence=8&isAllowed=y

[16] Arrow, KJ. The Economic Implications of Learning by Doing. The Review of Economic Studies. 1962; 29(3): 155–173. DOI: 10.2307/2295952

[17] Echeverría P, Mas Bergas MA, Puig J, Isnard M, Massot M, Vedia C, et al. COVIDApp as an Innovative Strategy for the Management and Follow-Up of COVID-19 Cases in Long-Term Care Facilities in Catalonia: Implementation Study. JMIR Public Health Surveill. 2020 Jul 17; 6(3): e21163. DOI: 10.2196/21163 32629425 PMC7373378

[18] Comas-Herrera A, Zalakaín J, Lemmon E, Henderson D, Litwin C, Hsu AT, et al. Mortality associated with COVID-19 in care homes: international evidence. Article in LTCcovid.org, International Long-Term Care Policy Network, CPEC-LSE, 1st February 2021. [cited 2023 31 August]. Available from: https://ltccovid.org/wp-content/uploads/2021/02/LTC_COVID_19_international_report_January-1-February-1-1.pdf

[19] Martínez-Payá M, Carrillo I, Guilabert M. Lessons Learned from the COVID-19 Pandemic in Nursing Homes: A Systematic Review. Int J Environ Res Public Health. 2022; 19: 16919. DOI: 10.3390/ijerph192416919 36554806 PMC9779143

[20] Byrd W, Salcher-Konrad M, Smith S and Comas-Herrera A. What Long-Term Care Interventions and Policy Measures Have Been Studied During the Covid-19 Pandemic? Findings from a Rapid Mapping Review of the Scientific Evidence Published During 2020. Journal of Long-Term Care. 2021; 423–437. DOI: 10.31389/jltc.97

[21] National Collaborating Centre for Methods and Tools. (2021, March 9). What strategies mitigate risk of COVID-19 outbreaks and mortality in long-term care facilities? Update 2. [cited 2023 31 August]. Available from: https://www.nccmt.ca/knowledge-repositories/covid-19-rapid-evidence-service

[22] National Collaborating Centre for Methods and Tools. (2022, July 18). Rapid Review: What COVID-19 testing requirements and/or recommendations do high-income countries currently have in place in healthcare and long-term care settings? [cited 2023 31 August]. Available from: https://nccmt.ca/pdfs/res/role-of-testing

[23] Fang FC, Benson CA, Del Rio C, Edwards KM, Fowler VG, Fredricks DN, et al. COVID-19-Lessons Learned and Questions Remaining. Clin Infect Dis. 2021; 72(12): 2225–2240. DOI: 10.1093/cid/ciaa1654 33104186 PMC7797746

[24] van Tol LS, Smaling HJA, Groothuijse JM, Doornebosch AJ, Janus SIM, Zuidema SU, et al. COVID-19 management in nursing homes by outbreak teams (MINUTES) — study description and data characteristics: a qualitative study. BMJ Open. 2021; 11: e053235. DOI: 10.1136/bmjopen-2021-053235 PMC8634633 34848521

[25] Notarnicola E, Perobelli E, Rotolo A, Berloto S. Lessons Learned from Italian Nursing Homes during the COVID-19 Outbreak: A Tale of Long-Term Care Fragility and Policy Failure. Journal of LongTerm Care. 2021; 221–229. DOI: 10.31389/jltc.73

[26] Pla de desplegament de l’atenció integrada social i sanitària a les persones que viuen a residències de gent gran. Barcelona: Direcció General de Planificació i Recerca en Salut; 2023 (in Catalan) [cited 2024 1 October]. Available from: https://hdl.handle.net/11351/10667

[27] Beogo I, Tchouaket EN, Sia D, Bationo NJ, Collin S, Tapp D, et al. Promising best practices implemented in long-term care homes during COVID-19 pandemic to address social isolation and loneliness: a scoping review protocol. BMJ Open. 2022; 12(1): e053894. DOI: 10.1136/bmjopen-2021-053894 PMC8724591 34980621

